# Visualize omics data on networks with Omics Visualizer, a Cytoscape App

**DOI:** 10.12688/f1000research.22280.2

**Published:** 2020-06-30

**Authors:** Marc Legeay, Nadezhda T. Doncheva, John H. Morris, Lars Juhl Jensen

**Affiliations:** 1 Novo Nordisk Foundation Center for Protein Research, University of Copenhagen, Copenhagen, Denmark; 2 Center for Non-Coding RNA in Technology and Health, Department of Veterinary and Animal Sciences, University of Copenhagen, Copenhagen, Denmark; 3 Resource for Biocomputing, Visualization and Informatics, University of California, San Francisco, USA

**Keywords:** Cytoscape, app, network visualization, omics data, network biology

## Abstract

Cytoscape is an open-source software used to analyze and visualize biological networks. In addition to being able to import networks from a variety of sources, Cytoscape allows users to import tabular node data and visualize it onto networks. Unfortunately, such data tables can only contain one row of data per node, whereas omics data often have multiple rows for the same gene or protein, representing different post-translational modification sites, peptides, splice isoforms, or conditions. Here, we present a new app, Omics Visualizer, that allows users to import data tables with several rows referring to the same node, connect them to one or more networks, and visualize the connected data onto networks. Omics Visualizer uses the Cytoscape enhancedGraphics app to show the data either in the nodes (pie visualization) or around the nodes (donut visualization), where the colors of the slices represent the imported values. If the user does not provide a network, the app can retrieve one from the STRING database using the Cytoscape stringApp. The Omics Visualizer app is freely available at https://apps.cytoscape.org/apps/omicsvisualizer.

## Introduction

Cellular functions are mediated by complex networks of interactions between genes, proteins, and other molecular entities. Omics technologies are commonly used to measure the detailed regulation of these networks by quantifying changes of individual post-translational modification (PTM) sites, peptides, or splice isoforms across different experimental conditions. However, it is not easy to visualize such data sets, which have multiple values per gene or protein, onto the networks using existing network visualization tools such as Cytoscape ^1^ or Gephi ^2^.

To address this, we present the new Omics Visualizer app for Cytoscape ^3^. The app allows users to import data tables with several rows referring to the same node and to visualize such data on networks; while designed with omics data in mind, the app is data agnostic. These values can be shown directly on the nodes of the networks as pie or donut visualizations, in which the color of each slice represents a different value for the same node. The Omics Visualizer app was implemented using the API that Cytoscape makes available to developers, and the data visualization builds upon the enhancedGraphics app ^4^. The app furthermore integrates with the stringApp ^5^ to facilitate easy visualization of data onto networks from the STRING database ^6^ and supports Cytoscape Automation to facilitate integration with other tools ^7^.

**Figure 1.**
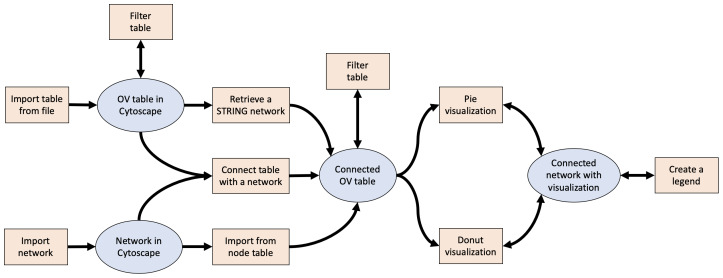
Workflow for visualizing data with Omics Visualizer. This workflow shows the three possible ways to obtain an Omics Visualizer table connected with a network, to then visualize the data with pie and/or donut visualizations.

The workflow for visualizing data with the app is outlined in Figure 1. The first key step is to obtain an Omics Visualizer table that is connected with a network in Cytoscape. There are three ways to do this: 1) import a table from a file and explicitly connect it to an existing network in Cytoscape, 2) import a table from a file and retrieve a STRING network based on the table, and 3) import selected columns from the node table of an existing Cytoscape network into an Omics Visualizer table. Once the table and the network are connected, it is possible to create data visualizations on the network and then generate a legend.

## Methods

### Implementation

The typical Omics Visualizer ^3^ workflow consists of four steps: importing data as a table, optionally filtering the data, connecting it to one or more networks, and finally visualizing the connected data onto the networks.

There are two ways to import an Omics Visualizer table, the first one is from a file, the second one is from a node table. The Omics Visualizer table import from file mimics the Cytoscape default import process. The app can handle text files (e.g. comma- or tab-delimited values) as well as spreadsheet files from e.g. Microsoft Excel. The app auto-detects the data type of each column, and the user can subsequently select which columns to import and modify the auto-detected data type for each column if needed. To also allow visualization of data from a network’s node table, selected columns can be imported as an Omics Visualizer table with three columns such that each row lists the identifier of a node ( *node* column), the name of a selected column from the node table ( *source* column), and the imported value ( *values* column). Both import methods create two private unassigned tables in Cytoscape: one that contains the data imported from the file, and another that stores all associated Omics Visualizer properties. These tables are private, which means that the user cannot interact with them directly in the Cytoscape UI. Instead, the data can be viewed (but not edited) through the Omics Visualizer panel located in the table panels. The tables are also accessible via the command interface and the Cytoscape API.

**Figure 2.**
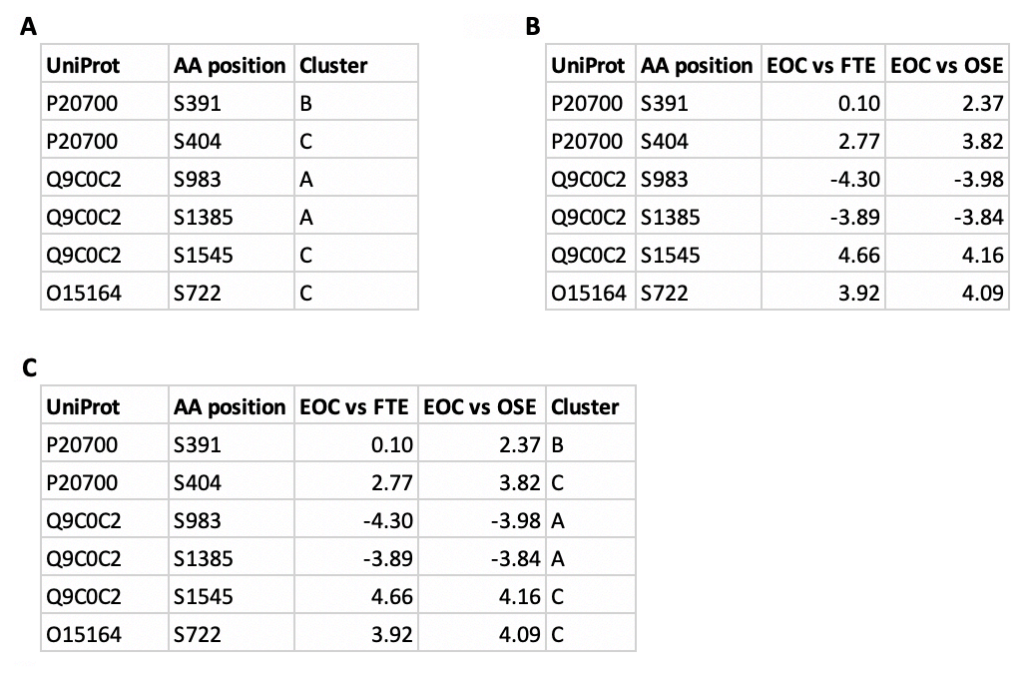
Desired formats for table import. This shows three examples of how tables can be formatted before being imported with Omics Visualizer. One column contains the node identifier (‘UniProt’), and the other columns contain the values to be visualized (‘Cluster’ with pie visualization, ‘EOC vs FTE’ and ‘EOC vs OSE’ with donut visualization). It is possible to format your table for pie visualization ( **A**), donut visualization ( **B**), or both ( **C**). You can have some extra columns that will not be used to visualize but for other purposes, for example, here the column ‘AA position’ identifies the specific site of the protein and are used as labels.

The imported file should be formatted as indicated in Figure 2, which is a sample of the table used in Figure 4 and Figure 5. One column must contain the node identifier value (‘UniProt’ in Figure 2), which will be used to connect the Omics Visualizer table with the node table. The other columns contain the data to be visualized. In case of a pie visualization, all values are in one column and the slices for one pie are contained in different rows with the same node identifier (‘Cluster’ column in Figure 2 A, C). In a case of donut visualizations, the values can be contained in several columns (‘EOC vs FTE’ and ‘EOC vs OSE’ columns in Figure 2 B, C). It is possible to have additional columns in the file, which can be imported and used to filter the rows, as protein identifiers for retrieving a STRING network or as labels (‘AA position’ in Figure 2).

If the data was not already filtered before importing it into Cytoscape, Omics Visualizer enables users to do so afterwards. Omics Visualizer can filter the rows based on selected columns with string, numeric and Boolean values and offers several operators depending on the data type: EQUALS, NOT_EQUALS, NULL and NOT_NULL for all types; CONTAINS, NOT_CONTAINS and MATCHES for string values; LOWER, LOWER_EQUALS, GREATER and GREATER_EQUALS for numeric values. Similar to the Cytoscape selection filters, Omics Visualizer interface allows the user to create Boolean formulas with nested criteria. Once the filter is applied, rows that do not satisfy the filter are hidden in the panel, and the active filter is stored in the properties table to allow it to be changed later.

To visualize the data table onto a network, the Omics Visualizer table must first be connected to the network by specifying matching key columns in the node and Omics Visualizer tables. This connection information is stored in the network table, which is used to recreate the link when a session file is loaded. An Omics Visualizer table can be connected to several networks, but a network can only be connected to one Omics Visualizer table. If the user wants to use both types of visualization provided by Omics Visualizer (pie and donut), the data for both visualizations should be imported as a single table ( Figure 2 C). A node table key can match the key in several Omics Visualizer table rows, in which case the node is connected to all of them. The number of connected rows for each node is stored in the node table; note that this number does not reflect any filtering. When the Omics Visualizer table is imported from the node table, the connection is automatically done using the identifier column ( *node* column) as key.

Alternatively, Omics Visualizer can retrieve and automatically connect a network from the STRING database ^6^ using the Cytoscape stringApp ^5^. The user can select any column with gene/protein identifiers, which will be used to first query STRING to retrieve a network and subsequently as the key for connecting the network to the Omics Visualizer table.

Once connected, the user can show the data from the Omics Visualizer table on networks using either a pie or a donut visualization. The data values are mapped to colors using either a discrete mapping, where the user chooses the colors for each value, or a continuous mapping, where the user defines a color gradient. For the continuous mapping, the user specifies three values and corresponding colors, namely the minimum, middle, and maximum. Every value lower than the minimum or greater than the maximum value will be shown using the minimum and maximum color, respectively. Predefined color mappings can be used with the help of Cytoscape palettes such as ColorBrewer ^8^ and Viridis (originally from Matplotlib ^9^). The charts are drawn thanks to the enhancedGraphics app ^4^. This app draws charts based on a description string, which is specified as a Cytoscape Custom Chart node style.

When the user creates a visualization, Omics Visualizer creates a column in the network table and several columns in the node table, and then it activates a Custom Chart node style: Omics Visualizer uses Custom Chart 7 to visualize pies and Custom Chart 8 to visualize donuts. The network table column is used to store the visualization properties. One node table column is created to store the enhancedGraphics string. Several node table columns are created to store the values of the visualizations: one column for pie visualization, and one column per ring for donut visualization. The values from the Omics Visualizer table rows are formatted into lists to fit the enhancedGraphics requirements. The enhancedGraphics continuous mapping always ranges between two defined values *minimum* and *maximum*, and a specific color can be given to zero. Omics Visualizer allows the user to define a minimum, middle and maximum value as it fits the data. Omics Visualizer centers the data around the middle value and adjusts the *minimum* and *maximum* accordingly. The values are only modified in the node table columns, not in the actual Omics Visualizer table.

Omics Visualizer can automatically generate legends from the visualizations in the form of Cytoscape annotations, which can be exported as part of the images if the user exports the network. The user may specify a title, font, font size and default location of the legend. To allow users to easily modify the legend, each element of the legend is a separate annotation. These are grouped to let the user move or delete the legend in one click, once the user has activated the Annotation Selection from Cytoscape (The “T” with a dotted border icon at the bottom of the network or the ‘Select → Mouse Drag Selects → Annotations Only’ menu). The name of the annotation group is used to differentiate the legend annotations from other annotations and should thus not be changed. When the legend is created, Omics Visualizer creates a network table column to store it.

All the columns created by Omics Visualizer in the node or network tables are in the namespace “Omics Visualizer”, enabling the user to easily identify and hide them if desired.

Omics Visualizer is built from Java 1.8 using Cytoscape API 3.7, meaning the minimum required version of Cytoscape is 3.7.0.

### Operation

Cytoscape can be used with R or Python through to Cytoscape Automation ^7^. Omics Visualizer implements commands in the specific ’ov’ namespace, allowing Omics Visualizer to be used with the REST API. A full documentation of the commands is available at https://github.com/marclegeay/omics-visualizer/blob/master/automation_documentation.md. With the commands, the users can import a table, filter it, connect it with an existing network, retrieve a STRING network, create visualizations, and generate legends.

## Use cases

We will illustrate how to use Omics Visualizer ^3^ by visualizing site-specific proteomics data from a phosphoproteomics study of ovarian cancer by Francavilla *et al.* ^10^. This study compares the phosphoproteome of primary cells derived from epithelial ovarian cancer (EOC) and two healthy tissues, namely ovarian surface epithelium (OSE) and distal fallopian tube epithelium (FTE). The goal of the study was to uncover cancer-specific changes in expression, phosphorylation state, and kinase signatures by comparing cancer and two healthy tissues (EOC vs. OSE, and EOC vs. FTE) for each site. The sites were then clustered into three clusters: A) sites abundant in healthy (OSE and FTE) but not cancerous tissues (EOC); B) sites abundant in FTE and EOC, but not OSE; and C) sites abundant only in cancerous tissue (EOC).

In this example, we want to visualize this site-specific data set on a network in which each node is a protein. We start from Table S3 from Francavilla *et al.*, where each line represents a phosphorylation site in a protein, as opposed to a protein. We will first load the table file into Cytoscape thanks to Omics Visualizer and then create a STRING network from the data. We will finally apply some style to the network to visualize the data.

### Importing the data from a file

Table S3 only contains the expression in the different samples, but not the individual comparisons between EOC and healthy tissues, so we modified it to add the comparison columns ’EOC vs OSE’ and ’EOC vs FTE’. The modified file is avialable as *Underlying data* ^11^.

To load the data, we will use the specific import feature from Omics Visualizer, that can be accessed from the ’File *→* Import *→* Omics Visualizer table from File...’ menu, or the ’Apps *→* Omics Visualizer *→* Import table from file’ menu. This opens a custom dialog, which is very similar to the standard Cytoscape import table dialog. The user can name the table (or a default name will be given) and select the columns and their type to import. By default, all columns are imported and their type is inferred according to the first hundred lines of the file.

Here, we specifically want to import the ’UniProt’ column to retrieve a STRING network afterwards, the ’AA Position’ column to label the sites, the comparison columns ’EOC vs OSE’ and ’EOC vs FTE’ with log-ratios to be visualized, the ’Adj p-value’ column to filter the table by significant sites, and the cluster assignment column ’Cluster’ to visualize it. The file can be loaded with the following command:


ov load file=
"/path/to/Francavilla2017CellRep.tsv"


**Figure 3.**
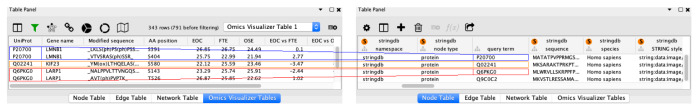
An Omics Visualizer table (left) and a node table (right) from a STRING network. Both tables can be linked together using the key column ‘UniProt’ from the Omics Visualizer table with the key column ‘query term’ from the node table. The color boxes identify the key values used to link the two tables, the color lines emphasize the link between the rows of the two tables.

Once the table is imported, the Omics Visualizer panel appears ( Figure 3, left table). The top part of the panel has a row of icons giving access to the different features, displays the number of rows of the table, and enables access to the different Omics Visualizer imported tables. The second part of the panel is the table itself.

**Figure 4.**
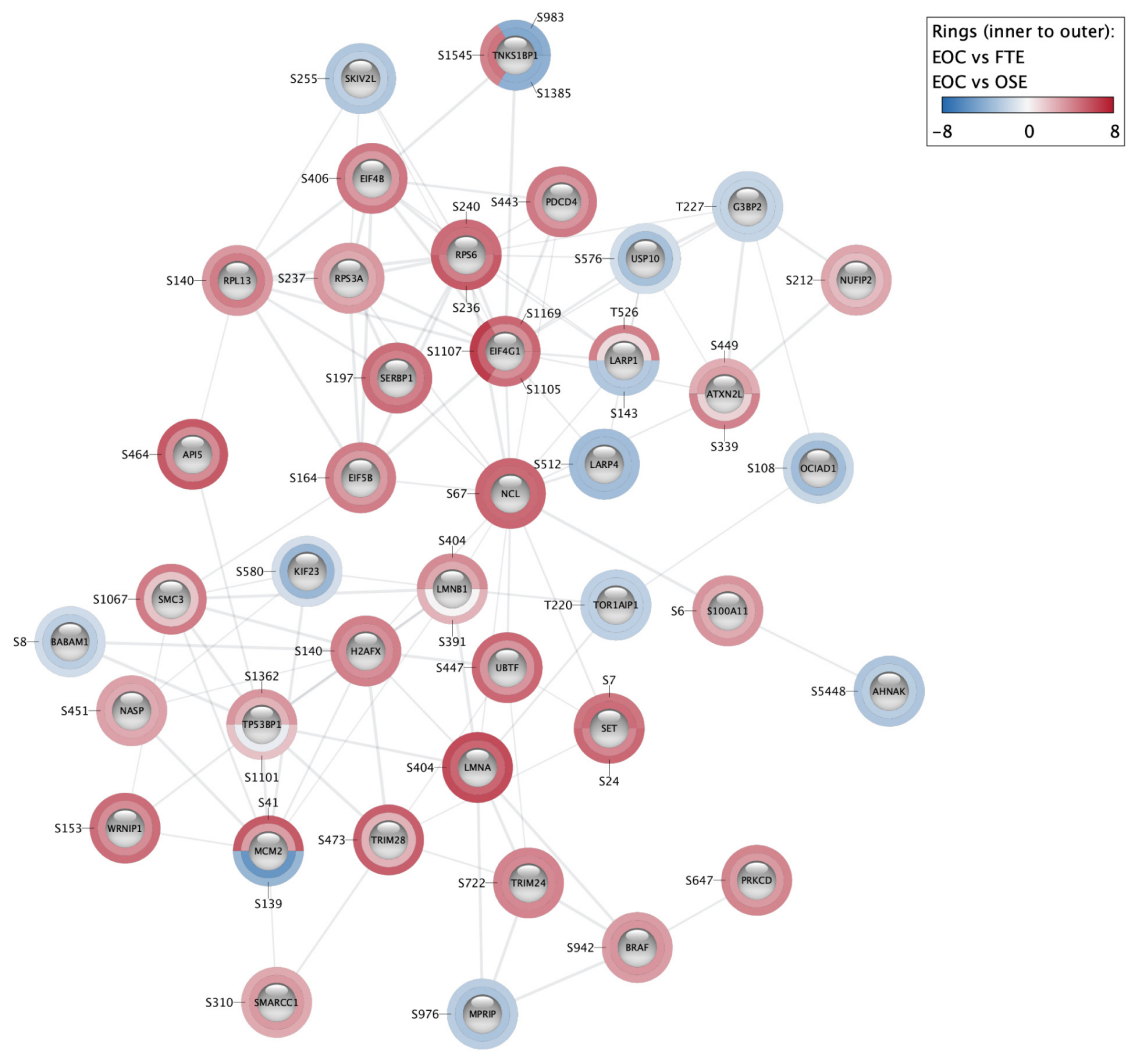
STRING network of proteins with significantly regulated phosphorylation sites detected in an ovarian cancer study ^10^. Log-ratios of protein abundance between cancer and healthy tissues were mapped around the nodes using a blue–white–red gradient. Each slice of the rings represents a significantly regulated phosphorylation site. The inner ring is the comparison between epithelial ovarian cancer and distal fallopian tube epithelium (EOC vs FTE), the outer ring is the comparison between epithelial ovarian cancer and ovarian surface epithelium (EOC vs OSE). This subnetwork is the second biggest cluster obtained using Markov clustering on the STRING network of all proteins from the study that have at least one phosphorylation site with an adjusted p-value lower or equal to 0.01.

**Figure 5.**
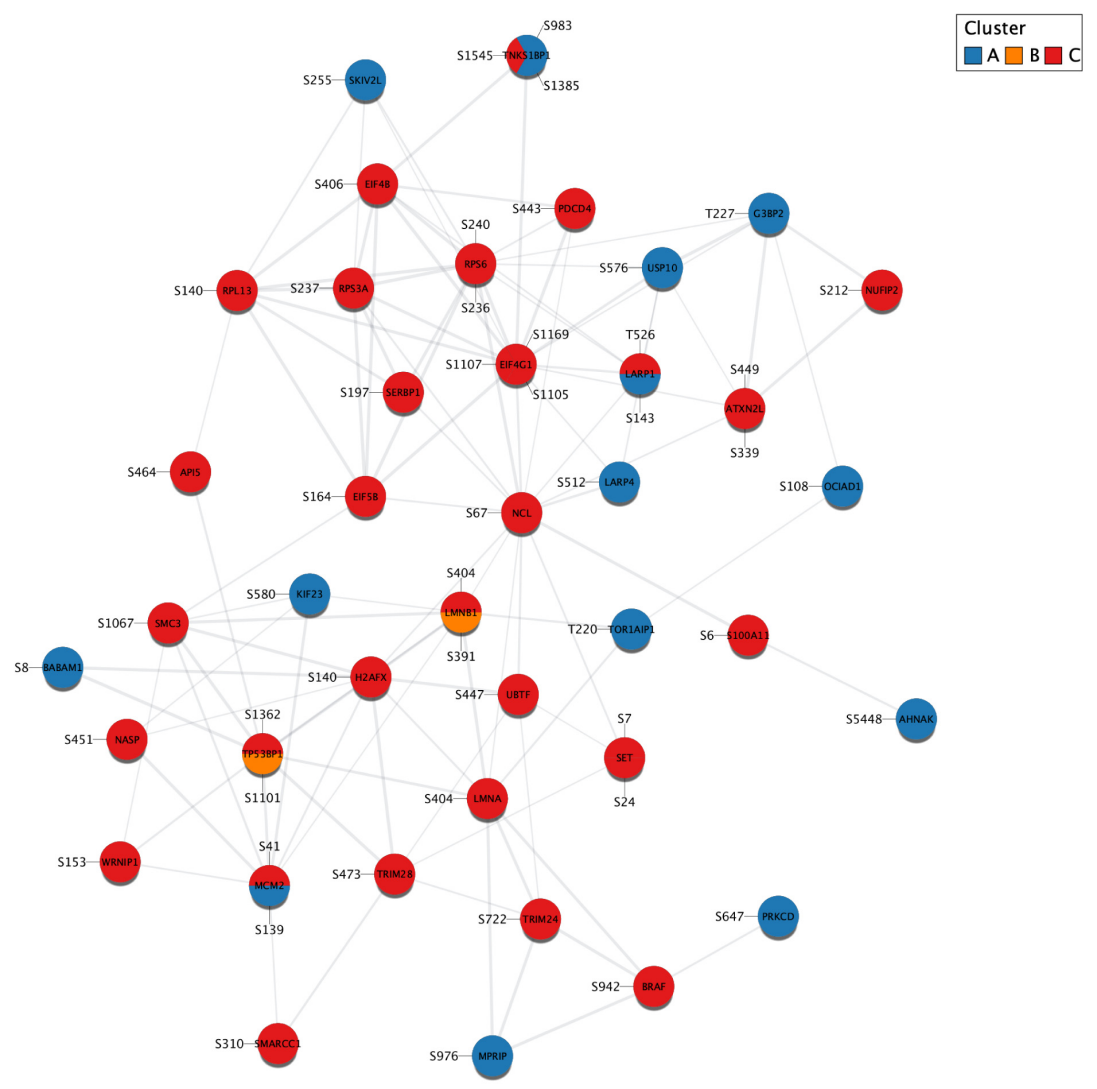
Same network as in Figure 4 with the clusters defined in the original study ^10^ mapped to the nodes. Each slice of a pie corresponds to a significantly regulated phosphorylation site of the protein and the color of the slice represents the cluster to which the site belongs. Sites abundant in healthy (OSE and FTE), but not cancerous (EOC) tissues are in cluster A; sites abundant in FTE and EOC, but not OSE are in cluster B; and sites abundant only in EOC are in cluster C.

### Filtering the table

It is possible to filter the table with the GUI or with the automation command. The filter GUI can be accessed *via* the filter icon from the Omics Visualizer panel ( Figure 3, left), or with the ’Apps *→* Omics Visualizer *→* Filter table’ menu. We filter the table so that we select only sites that have an adjusted p-value lower or equal to 0.01. This filter can be applied to the current table with the following automation command:


ov filter filter=
"(Adj p-value,LOWER_EQUALS,0.01)"


After the filter has been applied, the filter icon changes colors and the number of rows before and after filtering is displayed ( Figure 3, left table).

### Connecting a network

In this use case, we will use a STRING network corresponding to our data. With the help of the stringApp, we perform a ’protein query’ with the list of the UniProt identifiers of proteins from our table that have at least one phosphorylation site with an adjusted p-value lower or equal to 0.01.

The current version of the stringApp retrieves a STRING v11 network with default confidence of 0.4 that consists of 237 nodes and 1020 edges. To reduce the size of the network, we cluster it using the Markov clustering (MCL) from the clusterMaker2 app ^12^. We used the *inflation value* of 2.5 and the *stringdb score* as *array sources*. For illustrations purposes, we show only the second biggest cluster, which contains 40 nodes and 107 edges.

Once the STRING network is imported in Cytoscape by the stringApp, we connect the network with the table. The link icon from the Omics Visualizer panel ( Figure 3, left) or the ’Apps *→* Omics Visualizer *→* Manage table connections’ menu give access to the connect dialog. The dialog shows already connected networks and gives the possibility to modify or delete them. You can also create a new connection by selecting the network to connect to, then the two key columns from the network and from the table. In our case, the network was retrieved from the UniProt identifiers stored in the ’UniProt’ column of our table. The stringApp stores the query into the column ’query term’, so we use it as ’key column from Network’ and we use ’UniProt’ as the ’key column from Table’. Figure 3 illustrates the mapping between the two tables. The "P20700" (blue box) from the node table is linked to two rows of the Omics Visualizer table which have the same value in the UniProt column.

The connection between the current network and the current table can be performed with the following automation command:


ov connect mappingColNet="query term"
mappingColTable="UniProt"


### Retrieving a STRING network

It is possible to do the previous step more quickly by automatically retrieving a STRING network from the Omics Visualizer table. We can retrieve a STRING network with the STRING icon from the Omics Visualizer panel ( Figure 3, left) or the ’Apps *→* Omics Visualizer *→* Retrieve STRING network’ menu. We have to select the species, the column that contains the identifiers to query, the confidence cutoff and, if filtering was applied as in our case, whether to retrieve the identifiers only from filtered rows. By default the species is human, the cutoff is 0.40, and the query column is identified by a case-insensitive search for "uniprot" among the column names.

Once the STRING network is retrieved thanks to stringApp automation, the network is automatically connected to the Omics Visualizer table with the ’query term’ column from the node table with the query column selected by the user. The network can also be retrieved from the current Omics Visualizer table with the following automation command:


ov retrieve taxonID=9606 filteredOnly=true
queryColumn="UniProt"


### Donut visualization

The donut visualization can be configured *via* the donut icon from the Omics Visualizer panel ( Figure 3, left) or the ’Apps *→* Omics Visualizer *→* Create donut visualization’ menu.

Here, we want to visualize the two disease vs. healthy comparisons for each site. We select as *Values* the two comparison columns (EOC vs FTE and EOC vs OSE) that contain the numerical values we want to visualize. Because the values are log-ratios, we apply a continuous mapping and use a color scale. To be able to identify the individual phosphorylation sites in the network, we label the charts with the position of the amino acid (AA position column) of the site in the sequence of the protein. We customize the chart so that the first slice starts at a 3 o’clock position and the label font size is changed to 15. The next dialog enables the user to configure the color scale. By default, a diverging palette is selected, and the color scales from the minimum to the maximum value and is centered on zero. The minimum and maximum bound values are directly computed from the values of the table: the maximum bound is the maximum of the absolute value of the minimum and maximum data values; the minimum bound is the opposite of the maximum bound. The user can change both the values and the colors associated. In our case, we will change the range and set it from -8 to 8. If a table value is lower than the minimum value of the scale or larger than the maximum, the value will be associated with the color associated with the minimum or maximum value, respectively. The visualization can be modified or deleted by accessing the dialog again. The resulting network can be seen in Figure 4. We have two donuts around each node representing the two comparisons. One slice of a donut is one value from the Omics Visualizer table associated with the node: in our case, it is the log-ratio of a specific site. It is possible to flip the visualization, and have as many donuts as sites, with two slices for each comparison, by selecting “Ring is row” instead of the default “Ring is column” in the last dialog.

All the commands related to the donut visualization are associated with the keyword “outer” since the visualization is around the node. The visualization of the current table can be obtained with the following automation command:


ov viz apply outer continuous
attributes="EOC vs FTE,EOC vs OSE"
labels="AA position" filteredOnly=true
rangeMin=-8 rangeMax=8
chartSettings="arcstart:0,labelsize:15"


### Pie visualization

If we are not interested in the individual log-ratios from the comparisons, we can instead summarize the results as a pie visualization of the expression clusters.

The pie visualization can be configured *via* the pie icon from the Omics Visualizer panel ( Figure 3, left) or the ’Apps *→* Omics Visualizer *→* Create pie visualization’ menu. The pie visualization dialog looks like the donut visualization dialog, and the user must first select the column that contains the values to draw. The difference here is that the user can only select one column, because the different slices of the pie come from the different values associated with the same node. In our case, we want to visualize the "Cluster" column and apply a discrete mapping. As for the donut visualization, we will label the chart slices with the amino acid position (AA position). We also customize the chart to have the first slice starting at a 3 o’clock position and change the label font size to 15. The next dialog enables us to configure the color mapping. By default, Omics Visualizer selects a qualitative palette. Here the user may change the color associated with each value detected in the table. The default colors are too similar to each other and we changed them by clicking on the color square. The pie visualization can also be modified or deleted by accessing the dialog again.

All the commands related to the pie visualization are associated with the keyword “inner” since the visualization is inside the node. The resulting network is shown in Figure 5 and can be obtained with the following command:


ov viz apply inner discrete
attributes="Cluster"  labels="AA  position"
colorMapping="A:#1F78B4,B:#FF7F00,
C:#E31A1C" filteredOnly=true
chartSettings="arcstart:0,labelsize:15"


### Legend

The user can generate a legend by clicking the map icon from the Omics Visualizer panel ( Figure 3, left), or the ’Apps → Omics Visualizer → Manage legend’ menu, and choosing which legends to generate (pie and/or donut visualizations), the title, the font, and the position of the legend. Omics Visualizer will then create Cytoscape annotations corresponding to the current visualizations, displaying names of the columns above the color legends. In the case of the donut visualization, a list of column names shows the order of the columns. Because legends are annotations, they can be moved, edited, and exported with the network as an image. The user can modify an annotation by selecting it from the Annotation panel and choosing “Modify Annotation …” from the contextual menu. Note that the name of an annotation can be the same as its textual content, so the annotation needs to be modified for its content to change. To move an annotation, the user has to activate the Annotation selection (Select → Mouse Drag Selects → Annotations Only).

The legends in Figure 4 and Figure 5 were automatically generated with the following command:


ov legend draw position="EAST_TOP"
title=""


## Conclusions

Omics Visualizer ^3^ is a Cytoscape app that improves how omics data can be visualized on molecular networks. A key feature of Omics Visualizer is that, unlike the standard Cytoscape node table, it enables the user to import files with several rows of data related to the same node, with each row representing a different site, isoform, or experimental condition. This data can subsequently be visualized in or around the nodes as charts with a separate slice for each row that is connected to the node. These slices can be colored to show both continuous and discrete values, and Omics Visualizer can automatically produce a color legend to help in preparation of publication-ready figures. All the actions of Omics Visualizer can be done using the Cytoscape GUI or the commands, making it easy to use Omics Visualizer also in R or Python via the Cytoscape REST API.

## Data availability

### Underlying data

Zenodo: Modified Table S3 - Francavilla et al. 2017. https://doi.org/10.5281/zenodo.3629220 ^11^.

Data is available under the terms of the Creative Commons Attribution 4.0 International.

### Software availability

**The software, source code, and tutorial are available at the Cytoscape App Store:** http://apps.cytoscape.org/apps/omicsvisualizer.

**Archived source code at the time of publication:** https://doi.org/10.5281/zenodo.3631584 ^3^.

**License:** BSD 2-Clause "Simplified" License.
