# Supplementary material for: Visualize omics data on networks with Omics Visualizer, a Cytoscape App
Source: F1000Res. 2020 Jun 30;9:157. Originally published 2020 Feb 28. [Version 2] doi: 10.12688/f1000research.22280.2 (PMC7194485; doi:10.12688/f1000research.22280.2)
Supplement: Peer review history

# Peer review: Reviewer response for version 1

**DOI:** 10.5256/f1000research.24577.r61035

**Published:** 2020-04-15

**Authors:** Ruth Isserlin

**Affiliations:** 1 Donnelly Centre for Cellular and Biomolecular Research, Donnelly Centre for Cellular and Biomolecular Research, University of Toronto, Toronto, ON, Canada

Authors Legeay *et al.* introduce a new Cytoscape app to enable easy import and visualization of multi data type -omics data onto to networks with added features to quickly import interaction data from String. A great new addition to the cytoscape app set. 

**Detailed comments:**

**In the method section -**

 “the data can be viewed (but not edited) through the Omics Visualizer panel located next to the node, edge, and network table panels.” - not sure what you mean by this. Is it an additional tab in the table panel or is it its own panel next to the table pane? After running the app I see that it is an additional tab. it might sound better as “the data can viewed (but not edited) through the Omics Visualizer panel in the table panel.” (Just for aesthetics I really like it if the app icon can be included in the tab name as well. Makes it easier to spot and click on.)

 “but a network can only be connected to one Omics Visualizer table.” - Does this mean that you can’t annotate a network with isoform data and phosphorylations at the same time? You can use both pie and donut visualizations at the same time so as long as the different data types are represented in the same table you can use multiple types. Maybe specify that an network can only be associated with one omics set but if you integrate multiple omics data into one table prior to loading into cytoscape you can integrate different data types. 

 The paragraph starting with “To visualize the data table onto a network,” that outlines how the different tables are connected and the relationships of those connections would be much clearer with a figure outlining all the different connections described. 

 It would be helpful to have a description of the type of table the app is expecting and how the two different charts can be used. I.e. I loaded in a table with genes vs. Patients expecting to see all my patient data on the node but I think that it only maps one column for the pie chart and multiple columns for the donut so the expectation is that there will be duplicate genes in a given column for the pie.

 What is the maximum number of conditions/instances that can be mapped to an individual node and visualization type?

 “The enhancedGraphics continuous mapping always ranges from minimum to zero, and zero to maximum.” - are minimum values calculated based on the filtered set of -omics data or the entire dataset loaded?

 “Omics Visualizer can automatically generate legends from the visualizations in the form of Cytoscape annotations, which can be exported as part of the images if the user exports the network. “ - it was very difficult to find the legend. I had to come back to the text a few times to figure out that it was somewhere on the network canvas and compared to the network it was tiny so it was hard to find. 

**Use cases:**

 “To reduce the size of the network, we cluster it using the Markov clustering from the clusterMaker2 app” - in the clustermaker2 app Markov clustering is called MCL. Might be good to add MCL in brackets for users who don’t know they are equivalent.

 “For illustrations purposes, we here show only the second biggest cluster, which contains 40 nodes and 107 edges.” - it is not clear how you limit to just that cluster. Do you filter the network by __mclCluster or do you create a network of the clusters directly from the cluster maker2 app?

 “It is possible to flip the visualization, and have as many donuts as sites, with two slices for each comparison.” - how could this be done? Does it involve changing our choice of column to EOC and FTE and OSE separately?

 Inner and outer visualization can be confusing. At first I thought you were referring to the inner and outer rings of the donut. Maybe changing or adding Pie and Donut to Outer visualization and Inner visualization headers will be helpful.

 “The difference here is that the user can only select one column, because the different slices of the pie come from the different values associated with the same node. “ - This is an important distinction that needs to be specified earlier. Whether it is in the introduction or in the methods but I think that the different type of data Omics is expecting and the data types best used for each visualization types needs to be expanded on sooner in the paper.

 In the inner visualization section - “In our case, we want to visualize the "Cluster" column and apply a discrete mapping.” - it is unclear what the meaning for this “Cluster” column that was in the initial file loaded.

 In the legend section - “Because legends are annotations, they can be moved, edited, and exported with the network as an image.” - how can the legends be edited or moved? I tried right clicking on the legend and selected Edit -> modify annotation but nothing happened. I figured out that you modify the annotation through the annotation panel but might be good to add how you do it here. (I couldn’t figure out how to move it though)

**Minor grammatical suggestions:**

 “For illustrations purposes, we here show only the second biggest cluster, which contains 40 nodes and 107 edges.” - remove the “here” “For illustrations purposes, we show only the second biggest cluster, which contains 40 nodes and 107 edges.”

 “The visualization is configured thanks to a specific dialog” - not sure what this is saying.

 “The inner visualization can be configured thanks to the pie icon or the ’Apps → Omics Visualizer → Create pie visualization’ menu. “ - sounds better as “The inner visualization can be configured using the pie icon or the ’Apps → Omics Visualizer → Create pie visualization’ menu.”

Are the conclusions about the tool and its performance adequately supported by the findings presented in the article?

Yes

Is the rationale for developing the new software tool clearly explained?

Yes

Is the description of the software tool technically sound?

Yes

Are sufficient details of the code, methods and analysis (if applicable) provided to allow replication of the software development and its use by others?

Yes

Is sufficient information provided to allow interpretation of the expected output datasets and any results generated using the tool?

Yes

Reviewer Expertise:

bioinformatics

I confirm that I have read this submission and believe that I have an appropriate level of expertise to confirm that it is of an acceptable scientific standard.

# Response

**Published:** 2020-06-16

**Authors:** Marc Legeay

**Affiliations:** Novo Nordisk Foundation Center for Protein Research, Denmark

We would like to thank you for your comments and suggestions. We have addressed them in the new version of the manuscript, which also describes a new feature added to the app during review, namely to import columns from the node table of a network loaded in Cytoscape. We also added two new figures to describe the general Omics Visualizer workflow and to clarify the format needed for input tables.

 We took into account all your comments regarding the phrasing. For other comments, we give our answers below (we labeled your comments with “ **Reviewer**” and ours with “ **Response**”).

**Reviewer: **“but a network can only be connected to one Omics Visualizer table.” - Does this mean that you can’t annotate a network with isoform data and phosphorylations at the same time? You can use both pie and donut visualizations at the same time so as long as the different data types are represented in the same table you can use multiple types. Maybe specify that an network can only be associated with one omics set but if you integrate multiple omics data into one table prior to loading into cytoscape you can integrate different data types. 

**Response**: Right now, a network can only be connected to one Omics Visualizer table, so indeed if the user wants to have both visualizations, all the data has to be merged into one table (see new Figure 2C). We are currently working on a new version of the app that would enable a network to be connected to several tables.

**Reviewer: **It would be helpful to have a description of the type of table the app is expecting and how the two different charts can be used. I.e. I loaded in a table with genes vs. Patients expecting to see all my patient data on the node but I think that it only maps one column for the pie chart and multiple columns for the donut so the expectation is that there will be duplicate genes in a given column for the pie.

**Response**: We added a new Figure 2 giving three examples of tables that could be imported into Omics Visualizer. We hope it makes things easier to understand.

**Reviewer: **What is the maximum number of conditions/instances that can be mapped to an individual node and visualization type?

**Response**: There is no technical limit to the number, and it is thus up to the user to decide how many slices or rings to make. However, to create easily understandable visualizations, we would advise against having excessively many slices or rings.

**Reviewer: **“The enhancedGraphics continuous mapping always ranges from minimum to zero, and zero to maximum.” - are minimum values calculated based on the filtered set of -omics data or the entire dataset loaded?

**Response**: The user can put a minimum and maximum value, all values below the minimum will be colored as the minimum, and all values above the maximum will be colored as the maximum. Those values are, by default, the min and max of the filtered values. The color gradient from enhancedGraphics ranges from a negative value to zero, then zero to a positive value. Thus, Omics Visualizer has to change the table values to center the data. We modified the manuscript accordingly.

**Reviewer: **“Omics Visualizer can automatically generate legends from the visualizations in the form of Cytoscape annotations, which can be exported as part of the images if the user exports the network. “ - it was very difficult to find the legend. I had to come back to the text a few times to figure out that it was somewhere on the network canvas and compared to the network it was tiny so it was hard to find. 

**Response**: By default, the font size of the legend is the same as the font size of the node labels, meaning that if you have a huge network, the legend with default font size can be hard to find. When the legend is generated, we change the view of the network to fit everything (including the legend).

**Reviewer: **“For illustrations purposes, we here show only the second biggest cluster, which contains 40 nodes and 107 edges.” - it is not clear how you limit to just that cluster. Do you filter the network by __mclCluster or do you create a network of the clusters directly from the cluster maker2 app?

**Response**: We enabled the option “Create new clustered network” when we ran clusterMaker and extracted the second largest cluster by selecting all nodes and edges belonging to it and creating a new network from the selection.

**Reviewer: **“It is possible to flip the visualization, and have as many donuts as sites, with two slices for each comparison.” - how could this be done? Does it involve changing our choice of column to EOC and FTE and OSE separately?

**Response**: No, you can keep the same columns, it is just a matter of whether each ring represents a condition (“ring as column”) or a site (“ring as row”).

**Reviewer: **In the legend section - “Because legends are annotations, they can be moved, edited, and exported with the network as an image.” - how can the legends be edited or moved? I tried right clicking on the legend and selected Edit -> modify annotation but nothing happened. I figured out that you modify the annotation through the annotation panel but might be good to add how you do it here. (I couldn’t figure out how to move it though)

**Response**: We added an explanation on how to move and change the text of the legend using Cytoscape’s Annotations control panel.

# Peer review: Reviewer response for version 1

**DOI:** 10.5256/f1000research.24577.r60743

**Published:** 2020-03-10

**Authors:** Magnus Palmblad

**Affiliations:** 1 Center for Proteomics and Metabolomics, Leiden University Medical Center, Leiden, The Netherlands

To evaluate the user experience, I first downloaded and used the Omics Visualizer, following the on-line tutorial. The interface is intuitive (like how Cytoscape itself works), exposing just the right amount of information and options to the user. It is also clear to the user what the app does.

 The manuscript partially overlaps with but complements the on-line tutorial with background and some technical detail how the app integrates in Cytoscape. The Omics Visualizer fills a clear niche in the Cytoscape appverse, allowing tables to be imported surjectively (mapping multiple rows in the input to the same network node) as opposed to bijectively (with a one-to-one correspondence between the rows in the input table and nodes in the network). I suppose this could be achieved by manipulating tables (connecting row information in the data to columns in a node table) in R or Python, pushing the necessary data to Cytoscape through RCy3 or py2cytoscape (Omics Visualizer commands are also exposed via the Cytoscape REST API). But the Omics Visualizer is completely interactive and requires no scripting or formulae to be entered in the Cytoscape tables. The menus are clear and self-explanatory. The visualizer provides pie and donut visualizations for displaying multiple node attributes, such as post-translational modification sites and occupancy onto the same node. As would be expected from this team, Omics Visualizers also works well with the Cytoscape STRING app. I find this manuscript a very valuable companion to the app and on-line tutorial.

 The manuscript is very clearly written, and the length appropriate for the subject matter. I only have two minor comments:

1. "3 o’clock angle" should be "3 o’clock position" (in the "Outer" and "Inner visualization" paragraphs)?
2. The "Filter table rows" option referred to in the on-line tutorial is called "Filter table" in Cytoscape.

Are the conclusions about the tool and its performance adequately supported by the findings presented in the article?

Yes

Is the rationale for developing the new software tool clearly explained?

Yes

Is the description of the software tool technically sound?

Yes

Are sufficient details of the code, methods and analysis (if applicable) provided to allow replication of the software development and its use by others?

Yes

Is sufficient information provided to allow interpretation of the expected output datasets and any results generated using the tool?

Yes

Reviewer Expertise:

Analytical chemistry, molecular biology, mass-spectrometry based proteomics, bioinformatics.

I confirm that I have read this submission and believe that I have an appropriate level of expertise to confirm that it is of an acceptable scientific standard.

# Response

**Published:** 2020-06-16

**Authors:** Marc Legeay

**Affiliations:** Novo Nordisk Foundation Center for Protein Research, Denmark

We would like to thank you for your comments and we have modified the manuscript to take them into account. In the meantime, we also released a new version of the app with the additional feature to import columns from the node table of a network loaded in Cytoscape. We thus further modified the manuscript to describe the new feature. We also added two new figures to describe the general Omics Visualizer workflow and to clarify the format needed for input tables.
